# *Neisseria gonorrhoeae* molecular typing for understanding sexual networks and antimicrobial resistance transmission: A systematic review

**DOI:** 10.1016/j.jinf.2018.02.011

**Published:** 2018-06

**Authors:** Katy Town, Hikaru Bolt, Sara Croxford, Michelle Cole, Simon Harris, Nigel Field, Gwenda Hughes

**Affiliations:** a National Institute for Health Research Health Protection Research Unit in Blood Borne and Sexually Transmitted Infections at University College London in partnership with Public Health England and in collaboration with the London School of Hygiene and Tropical Medicine, Mortimer Market Centre, Third Floor, Capper Street, London WC1E 6JB, UK; b Centre for Molecular Epidemiology and Translational Research, Institute for Global Health, University College London, Mortimer Market Centre, Capper Street, London WC1E 6JB, UK; c HIV/STI Department, National Infection Service, Public Health England, 61 Colindale Avenue, London NW9 5EQ, UK; d Antimicrobial Resistance and Healthcare Associated Infections (AMRHAI) Reference Unit, National Infection Service, Public Health England, 61 Colindale Avenue, London NW9 5EQ, UK; e The Wellcome Trust Sanger Institute, Genome Campus, Cambridge CB10 1SA, UK

**Keywords:** Neisseria gonorrhoeae, Sexually transmitted infection, Gonorrhoea, Molecular epidemiology, Molecular typing, Whole genome sequencing, Neisseria Gonorrhoeae Multi-Antigen Sequence Typing, Multi-Locus Sequence Typing, Public health, Sexual health

## Abstract

**Objectives:**

*Neisseria gonorrhoeae* (NG) is a significant global public health concern due to rising diagnoses rates and antimicrobial resistance. Molecular combined with epidemiological data have been used to understand the distribution and spread of NG, as well as relationships between cases in sexual networks, but the public health value gained from these studies is unclear. We conducted a systematic review to examine how molecular epidemiological studies have informed understanding of sexual networks and NG transmission, and subsequent public health interventions.

**Methods:**

Five research databases were systematically searched up to 31st March 2017 for studies that used sequence-based DNA typing methods, including whole genome sequencing, and linked molecular data to patient-level epidemiological data. Data were extracted and summarised to identify common themes.

**Results:**

Of the 49 studies included, 82% used NG Multi-antigen Sequence Typing. Gender and sexual orientation were commonly used to characterise sexual networks that were inferred using molecular clusters; clusters predominantly of one patient group often contained a small number of isolates from other patient groups. Suggested public health applications included using these data to target interventions at specific populations, confirm outbreaks, and inform partner management, but these were mainly untested.

**Conclusions:**

Combining molecular and epidemiological data has provided insight into sexual mixing patterns, and dissemination of NG, but few studies have applied these findings to design or evaluate public health interventions. Future studies should focus on the application of molecular epidemiology in public health practice to provide evidence for how to prevent and control NG.

## Highlights

• Combined molecular and epidemiological data can describe the spread of gonorrhoea.
• Sexual networks can be inferred from molecular clusters of infection.
• Gender and sexual orientation are commonly used to characterise these networks.
• Application of these data within gonorrhoea control interventions is limited.
• Future studies should focus on evaluating molecular typing data in practice.

## Introduction

*Neisseria gonorrhoeae* is a sexually transmitted pathogen of significant public health concern due to rising diagnosis rates, particularly in men who have sex with men (MSM), and the emergence of resistance to all classes of antimicrobials used for treatment.^1^, ^2^ It is important to better understand how and why *N. gonorrhoeae* and resistant infections spread within sexual networks in order to design targeted and rational interventions to control transmission.

Traditional epidemiological methods using surveillance data and patient questionnaires of reported behaviour to understand and describe sexual networks have been used to improve our understanding of the distribution and transmission of *N. gonorrhoeae*, including the concentration of *N. gonorrhoeae* in specific groups at risk.^3^, ^4^ However, these methods are limited in their ability to determine whether infected individuals with the same epidemiological characteristics are part of the same transmission network. Establishing direct epidemiological links is challenging because gonorrhoea is frequently asymptomatic, especially in females, which may lead to missing (undiagnosed) cases in most datasets.

Molecular technologies can be used to group isolates according to similarities in their genetic data and infer relatedness between isolates.^5^ By combining these molecular data with epidemiological data, hypotheses about the transmission of *N. gonorrhoeae* within and across different sexual networks can be tested. The use of molecular epidemiology in the analysis of *N. gonorrhoeae* populations is a rapidly evolving field but there has been no systematic assessment of its public health value.

In this systematic review, we aimed to use published literature to investigate how linked *N. gonorrhoeae* epidemiological and molecular typing data can enhance our understanding of sexual networks and pathogen transmission and how this information has been used within public health interventions to control gonorrhoea infections.

## Methods

### Search strategy and selection criteria

In this systematic review (reported according to the international prospective register of systematic reviews: PROSPERO 2016 CRD 42016037238),^6^ studies were included if sequence based DNA typing methods were used (Multi-locus Sequence Typing (MLST), *N. gonorrhoeae* Multi-Antigen Sequence Typing (NG-MAST) and Whole Genome Sequencing (WGS)) (see Supplementary material for Glossary of Terms) and the typing data were linked to patient-level epidemiological data, including patient demographic data (e.g. gender, age, and ethnicity), sexual behaviour data (e.g. sexual orientation, sex work, condom use, number of partners, sex abroad) and/or clinical data (e.g. symptoms, site of infection, concurrent STIs, HIV status). We focused on the sequence-based DNA typing techniques described above as these are currently the most commonly used and recommended^7^ techniques for molecular epidemiological studies. Studies were excluded if the articles were not in English, if the typing data were only linked to the geographical location and/or date of the isolates and not to patient epidemiological data, if typing data were only used to investigate the pathobiology of *N. gonorrhoeae*, or to develop methods for typing of *N. gonorrhoeae*.

We searched Web of Science, Scopus, MEDLINE, EMBASE, and the Cochrane library from inception to 31st March 2017. Search terms included the typing methods of interest (NG-MAST, MLST, and WGS) and terms related to *Neisseria gonorrhoeae*. The full search strategies are detailed in the Supplementary material, including details of conference abstract books that were searched for relevant studies.

The title, abstract and then full text of studies were assessed against the eligibility criteria. Where no full text was found, study authors were contacted by email to request a copy of the paper. Authors of conference abstracts were also contacted for the poster or presentation slide set.

### Data extraction and synthesis

Data were extracted using a standardised form and summarised in a descriptive table. Common themes were identified and are presented using a narrative approach. Risk of bias was assessed considering the potential for (1) selection bias affecting the interpretation and generalisability of results based on the isolates with typing data available and the wider population that the study aimed to represent, (2) missing data bias, and whether data were likely to be missing at random or not, and (3) reporting bias, particularly for reported behavioural variables.

### Dual independent review

Each article was reviewed, data extracted and risk of bias assessed independently by two reviewers (KT and HB, or KT and SC); disagreements were resolved at a meeting between the reviewers.

## Results

### Article screening

**Fig. 1:**
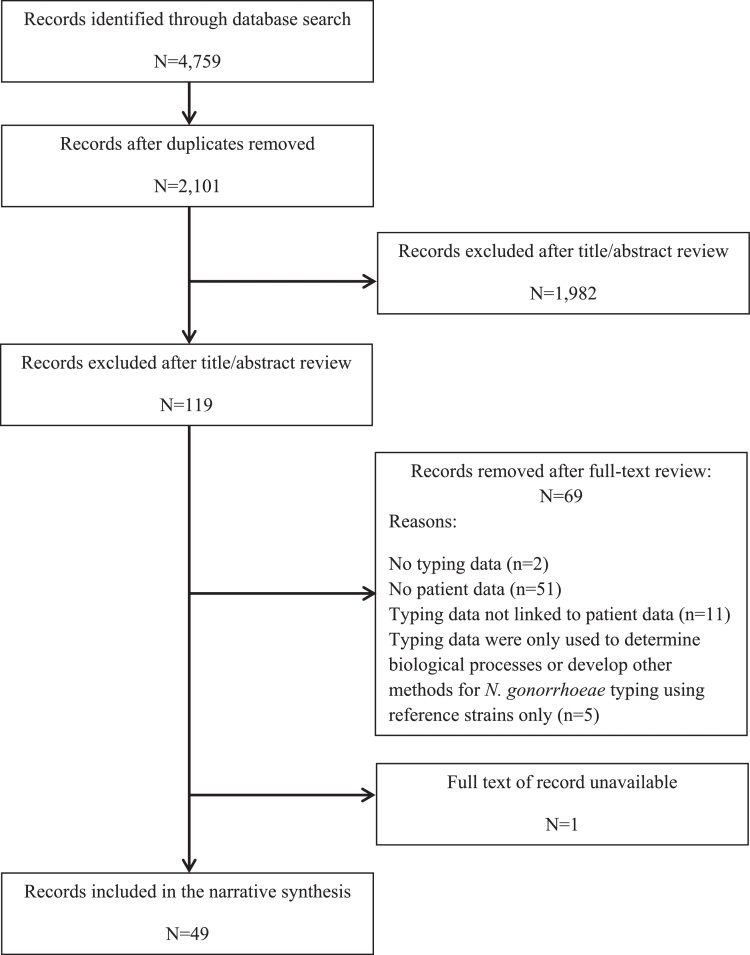
PRISMA flowchart indicating the systematic selection of journal articles for inclusion in this review PRISMA flowchart for the conference abstract book is presented in the Supplementary material.

Our search strategy identified 4759 studies, of which 2101 were unique (Fig. 1). Most studies were excluded following title and abstract review (94%; 1982/2,101). Of the remaining 119 studies, 50 met the study eligibility criteria (42%; 50/119). One study was not reviewed as the full-text could not be accessed.^8^ Studies were most commonly excluded at this stage because no patient data were reported (74%; 51/69). Nine conference abstracts were identified, four of which had been subsequently published. Authors for the remaining five were contacted for the presented poster or slide set but none replied (Supplementary material). Hence, 49 studies were included in this systematic review.

### Description of included studies

Over one third of studies used isolates collected in the UK (37%; 18/49),^9^, ^10^, ^11^, ^12^, ^13^, ^14^, ^15^, ^16^, ^17^, ^18^, ^19^, ^20^, ^21^, ^22^, ^23^, ^24^, ^25^, ^26^ from consecutive patients during the study time period (35%; 17/49),^11^^,^^14^, ^15^, ^16^, ^17^^,^^21^, ^22^^,^^24^^,^^27^, ^28^, ^29^, ^30^, ^31^, ^32^, ^33^, ^34^, ^35^ or limited inclusion of isolates to those known to be resistant to a specific antimicrobial (35%; 17/49).^9^, ^10^^,^^13^^,^^18^, ^19^, ^20^^,^^23^^,^^36^, ^37^, ^38^, ^39^, ^40^, ^41^, ^42^, ^43^, ^44^, ^45^ The number of isolates sampled ranged from six to 3326, with studies covering a time period of less than six months to nine years (Supplementary material).

**Fig. 2:**
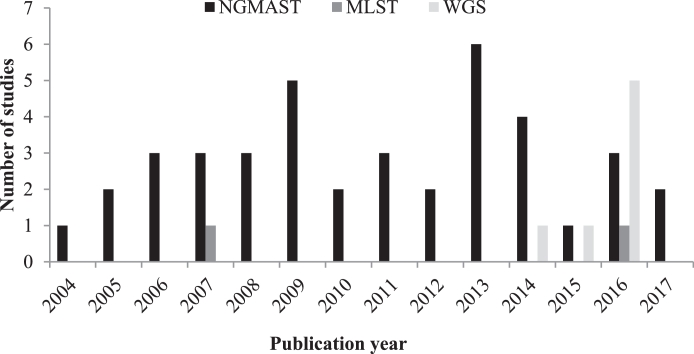
Number of studies by typing method over time (*N* = 49).

Most studies used NG-MAST (82%; 40/49),^9^, ^10^, ^11^, ^12^, ^13^, ^14^, ^15^, ^16^, ^17^, ^18^, ^19^, ^20^, ^21^, ^22^^,^^26^, ^27^, ^28^, ^29^, ^30^, ^31^, ^32^, ^33^^,^^35^, ^36^, ^37^, ^38^, ^39^, ^40^, ^41^, ^42^, ^43^, ^44^^,^^46^, ^47^, ^48^, ^49^, ^50^, ^51^, ^52^, ^53^ two used MLST (4%; 2/49),^34^, ^54^ and seven used WGS (14%; 7/49),^23^, ^24^, ^25^^,^^45^^,^^55^, ^56^, ^57^ (Fig. 2). The WGS studies also determined the NG-MAST and/or MLST sequence type in silico and compared typing methods. Although the clustering identified through NG-MAST and MLST methods broadly correlated with the clustering determined by WGS-based phylogenetic analyses, the WGS analyses showed that the other methods sometimes misclassified clustering. For example, Didelot et al*.*^25^ showed that isolates with an identical NG-MAST type that occurred within the same time period and location, and which would otherwise have been considered to be part of the same sexual network, were actually genetically distinct when WGS was used.

### Risk of bias

Most studies applied specific selection criteria to the isolates chosen for typing, which mainly related to the setting and location isolates were selected from, the time period for data collection, or the selection of isolates with a specific antimicrobial susceptibility profile. This may limit the generalisability of the results. Most studies typed ≥ 70% of isolates eligible under the predetermined selection criteria of the study. The main reasons not all isolates were typed were that the isolate could not be retrieved or the typing method failed. In general missing epidemiological data were minimal. However, most studies did not define how patient-level variables were collected, and it was therefore often difficult to assess reporting bias. It is likely that variables related to sexual behaviour, such as sexual orientation, were self-reported, which increases the risk of reporting bias. Further details for each study are presented in the Supplementary material.

### Patient-level epidemiological data

**Fig. 3:**
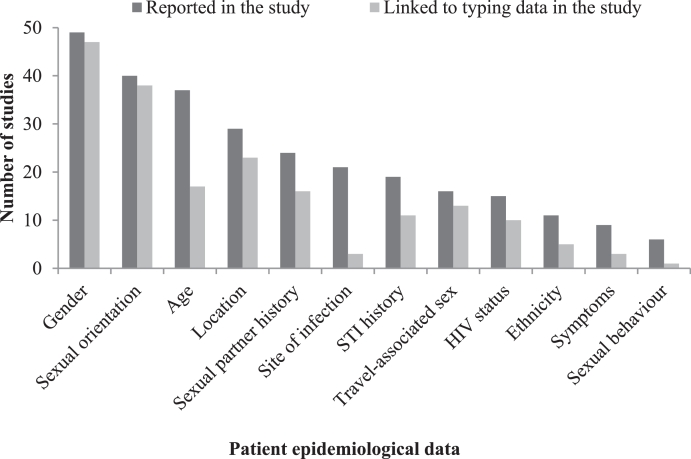
Reporting and linkage of patient epidemiological data to typing data (*N* = 49).

The most commonly reported and linked patient-level epidemiological data were gender (reported 100%; 49/49^9^, ^57^, linked 90%; 44/49^9^, ^11^, ^44^, ^46^, ^49^, ^53^, ^57^) and sexual orientation (reported 82%; 40/49^9^, ^11^, ^13^, ^23^, ^25^, ^30^, ^32^, ^33^, ^36^, ^42^, ^45^, ^53^, ^55^, ^57^, linked 71%; 35/49^9^, ^11^, ^13^, ^23^, ^25^, ^30^, ^32^, ^33^, ^36^, ^42^, ^46^, ^49^, ^53^, ^55^, ^57^). Other patient-level epidemiological data (not always linked to molecular data) included age, sexual partner history (such as the number of sexual partners), location of patient residence or clinic, site of infection, travel associated sex, STI history, patient HIV status, patient ethnicity, infection symptoms and sexual behaviour (Fig. 3).

### Summary of study findings

#### Identifying clusters of infection, sexual networks and transmission links

The majority of studies that used NG-MAST or MLST defined a cluster by the presence of two or more isolates with identical sequence types.^9^, ^10^^,^^16^, ^29^^,^^31^, ^42^ Closely related sequence types (defined as varying genetically by ≤ 1%) were sometimes grouped into larger categories called genogroups based on sequence similarity. Most studies identified a large number of different sequence types: many of which had not been identified before (‘novel’ sequence types) and/or were present only once in the isolate set (‘unique’ sequence types).^29^, ^30^, ^31^^,^^53^ For example, Chisholm et al.^29^ found 406 NG-MAST sequence typesamongst 1066 isolates. Over half of these were novel (216/406) and/or unique (281/406). WGS studies used maximum likelihood or Bayesian phylogenetic statistical modelling techniques with information on the number of single nucleotide polymorphisms (SNPs) to identify similarities and allocate isolates into clusters.

The molecular data were also used to provide evidence about the likelihood that transmission of *N. gonorrhoeae* had occurred between two patients.^9^, ^11^^,^^12^, ^14^^,^^16^, ^17^^,^^23^, ^24^, ^25^^,^^38^, ^53^ The degree of concordance between sexual contact pairs (confirmed through partner notification data) and genetic data, such as NG-MAST sequence type, was > 85% in these studies.

#### Describing sexual networks using typing and patient-level epidemiological data

Patient-level epidemiological data were used to characterise and compare clusters of infection (Fig. 3). For example, Horn et al.^31^ found that isolates classified as genogroup 25 were predominantly from women, whereas those classified as genogroup 1407 were predominantly from men. In general, only the most common sequence types, which formed the largest clusters, were described in this way.^10^, ^11^^,^^15^, ^16^, ^17^, ^18^, ^19^^,^^21^, ^27^^,^^31^, ^32^^,^^40^, ^47^^,^^48^, ^50^^,^^53^ Where sequence types were statistically associated with one group of patients, such as MSM, authors inferred that this provided evidence of a discrete sexual network. Further examples are described below:

(i) Sexual orientation:

Overall, 38 studies investigated the relationship between sexual orientation and *N. gonorrhoeae* sequence type. Most (87%; 33/38) identified a difference in the sequence types circulating between MSM and heterosexual patients. For example, Cole et al.^16^ found a statistically significant association between NG-MAST sequence types and sexual orientation: sequence types 147, 4, 1634 and 64 were more likely to be from MSM than heterosexual patients. However, often, clusters with a predominating patient characteristic also contained a small number of isolates from other patient groups. For example, Wong et al.^32^ found that NG-MAST sequence type 547 was associated with MSM but four of the 16 isolates were from heterosexual men. There was evidence of the same sequence type being associated with different sexual orientation characteristics in other studies. For example, using isolates collected in 2003, Abu-Rajab et al*.*^11^ found that NG-MAST sequence type 210 was associated with heterosexuals in Glasgow, Scotland. A year later, in 2004, Choudhury et al*.*^15^ found the same NG-MAST sequence type was associated with MSM in London, England.

(ii) Sub-groups within MSM and heterosexual networks:

Half of the studies that included patient sexual orientation (17/38) investigated the existence of discrete transmission clusters within larger sexual networks by separately analysing the association between sequence types and patient variables within these sub-groups of the population. This enhanced analysis helped describe clusters of infection in more detail. For example, Choudhury et al*.*^15^ found that isolates from heterosexual patients had NG-MAST sequence types that varied by ethnicity and age, indicating discrete sexual networks, but they did not identify similar patterns associated with specific characteristics in MSM. In contrast, Bernstein et al*.*^27^ found that some NG-MAST sequence types were associated with specific characteristics in MSM. For example, isolates classified as NG-MAST sequence type 2992 were more likely to be from MSM who reported oral sex only, and other NG-MAST sequence types varied by reported sexual risk behaviour (three or more sexual partners compared to fewer sexual partners).

(iii) HIV status:

There were 10 studies that compared patient HIV status to the typing data to investigate whether the molecular data provided evidence of discrete sexual networks in people with and without HIV.^15^, ^16^, ^17^^,^^27^, ^32^^,^^42^, ^46^^,^^47^, ^50^^,^^53^ Not all studies found the same result, which is likely due to the different populations and sexual behaviours of these populations investigated in each study. For example Didelot et al*.*^25^ found that *N. gonorrhoeae* was more likely to be transmitted between two HIV-positive patients than between a HIV-positive and HIV-negative patient, suggestive of serosorting sexual behaviour (the term for engaging in condomless anal intercourse with partners of the same known or presumed HIV status).^58^ However, Bernstein et al*.*^27^ and Cheng et al*.*^47^ did not find an association between molecular clusters and HIV status.

(iv) Travel-associated sexual partnerships:

In 13 studies^9^, ^10^, ^11^^,^^13^, ^15^^,^^16^, ^19^^,^^20^, ^24^^,^^25^, ^28^^,^^30^, ^43^, the molecular typing data was used to describe and investigate the role of travel-associated sexual partnerships on *N. gonorrhoeae* transmission and acquisition. Some studies found that unique sequence types were more likely to have been acquired outside of the local area, whereas clustered isolates were more likely to have been acquired within the local area^10^, ^15^^,^^19^, ^20^. Fernando et al*.*^17^ found that patients attending a specialist STI clinic in Edinburgh, Scotland, infected with unique *N. gonorrhoeae* NG-MAST sequence types were more likely to have reported recent sexual contacts from outside the local area. Similarly, Martin et al.^10^ found that the common strains types identified in London, England, were more likely to be from patients who did not report sex abroad.

#### Describing antimicrobial resistance (AMR) within sequence types

To describe and identify sequence types of *N. gonorrhoeae* associated with AMR, phenotypic antimicrobial susceptibility data were combined with molecular typing data in 83% of studies (41/49). These studies found that susceptible *N. gonorrhoeae* were genetically more diverse than resistant *N. gonorrhoeae*, as evidenced by the higher number of different NG-MAST sequence types in the susceptible clones*.*^36^, ^49^^,^^54^ Non-susceptible *N. gonorrhoeae* tended to be more clonal, for example, *N. gonorrhoeae* with decreased susceptibility to cefixime was usually detected in isolates identified as the NG-MAST sequence type 1407.^13^, ^18^^,^^30^, ^31^^,^^36^, ^37^^,^^48^, ^53^ Similar clonality of the gonococcal population was found for other antimicrobial resistant phenotypes, such as ciprofloxacin, penicillin and tetracycline. Azithromycin resistance was found to occur sporadically in the gonococcal population rather than be associated with a particular clone,^42^, ^55^ with the exception of high-level azithromycin resistant isolates (minimum inhibitory concentration > 256 mg/L) that were found in clusters of closely related gonococci*.*^14^, ^23^^,^^35^, ^44^

When the phenotypic data, molecular data and patient-level epidemiological data were all combined, this provided more insight into the distribution and spread of resistant *N. gonorrhoeae* infection in sexual networks or across populations.^10^, ^14^^,^^21^, ^27^^,^^29^, ^47^^,^^50^, ^56^^,^^57^ For example, using WGS, Grad et al*.*^57^ reported that *N. gonorrhoeae* with decreased susceptibility to cefixime first appeared on the west coast of the United States and then spread eastwards primarily within MSM networks, but with a small number of diagnoses in heterosexual patients. Authors often compared their findings to other studies to assess whether there were sexual networks operating between cities or countries and how this contributed to the spread of resistant infection. For example, Chisholm et al.^14^ speculated that high-level azithromycin-resistant *N. gonorrhoeae* (MIC ≥ 256 mg/L) identified in Liverpool, England, was imported from Scotland because the NG-MAST sequence types (ST649), AMR phenotype and sexual orientation of the infected patients matched those of cases previously reported by Palmer et al.^21^

#### Public health application of findings from included studies

Many studies stated that combining molecular typing data and patient-level epidemiological data could be used to support decisions about *N. gonorrhoeae* prevention and control activities (Table 1). Over half of the studies (54%; 26/49)^9^, ^10^, ^11^^,^^15^, ^16^, ^17^, ^18^, ^19^, ^20^, ^21^, ^22^^,^^24^, ^25^^,^^27^^,^^32^, ^33^, ^34^^,^^39^, ^40^, ^41^^,^^44^, ^50^^,^^51^, ^56^^,^^57^ suggested that these data could be used to identify which patient groups to target for public health interventions in order to maximise the effectiveness of resources. Other public health uses of the typing data included confirming outbreaks of new strains, including AMR infection,^9^, ^20^^,^^23^, ^26^^,^^52^ or using the data to evaluate existing public health interventions,^9^, ^10^^,^^22^, ^30^^,^^49^ such as confirming the success of partner notification in a population. For example, Monfort et al. concluded that the high number of single STs in their sample was probably due to the lack of effective contact tracing. Similarly, many studies (22%; 11/49)^11^, ^12^^,^^15^, ^16^^,^^19^, ^22^^,^^24^, ^25^, ^26^^,^^51^, ^57^ suggested that the molecular data could support contact tracing by providing complementary or confirmatory information.

**Table 1:** Potential uses of molecular epidemiology to support or evaluate public health interventions suggested by reviewed studies.

Public health intervention	Potential use of molecular epidemiology
Outbreak investigation	- the similarity between NG DNA has been used to confirm whether cases are related and potentially part of an outbreak^23^, ^26^^,^^52^
	- modelling using WGS has been used to estimate the number of undetected cases in an outbreak^25^
Sexual partner tracing	- as with outbreak investigation, genetic similarities or differences between NG isolates might be used to inform sexual partner tracing and complement partner notification data^11^, ^12^^,^^15^, ^16^^,^^19^, ^22^^,^^24^, ^25^^,^^51^, ^57^
	- identifying likely transmission between isolates using WGS data has been used to determine the density of sexual networks and speed of transmission^24^, ^25^
Antibiotic selection for patient management	- associations found between specific sequence types and antimicrobial resistance may be used to guide patient treatment where phenotypic resistance data are unavailable or delayed^11^, ^16^^,^^34^, ^42^^,^^45^, ^55^
Targeted health promotion/behavioural interventions	- associations found between specific sequence types and sub-groups of the population might help to identify the sexual network and determine groups for targeting with specific public health interventions^9^, ^11^^,^^16^, ^17^^,^^19^, ^23^^,^^29^, ^32^^,^^34^, ^36^^,^^37^, ^42^^,^^45^, ^46^, ^47^^,^^55^
	- the associations found between specific sequence types and sub-groups of the population might be useful for identifying and possibly quantifying the mixing between different groups, such as MSM and heterosexuals, which may help estimate the effect of tailored public health messages to one group vs the whole population^15^, ^16^
	- STI testing recommendations may be tailored based on whether the NG strain is associated with coinfection with another STI, including HIV^11^, ^17^^,^^24^, ^27^

NG = *Neisseria gonorrhoeae,* MSM = men who have sex with men.

Over one third of studies (39%; 19/49)^9^, ^11^^,^^16^, ^17^^,^^19^, ^23^^,^^26^, ^27^, ^28^, ^29^^,^^32^, ^34^^,^^36^, ^37^^,^^42^^,^^45^, ^46^, ^47^^,^^55^ suggested the information could be used to tailor the clinical management that individual patients receive. For example, if a patient is infected with a strain associated with AMR, an ongoing outbreak or coinfection with another STI, they might be provided with enhanced clinical care, either informing antimicrobial choice or prioritising the patient for test-of-cure and partner notification. Several studies^23^, ^33^ highlighted the importance of undertaking localised and prospective molecular epidemiological studies in order to identify changing trends in sequence types that may be indicative of new gonorrhoea outbreaks.

## Discussion

This systematic review identified 49 studies between 2004 and 2017 that used molecular typing data linked to patient-level epidemiological data to describe sexual networks of *N. gonorrhoeae* and AMR infection. NG-MAST was the most frequently used typing method, but WGS has become increasingly popular in more recent years. These molecular data have informed our understanding about the mutability of the *N. gonorrhoeae* genome, the relationship between genotype and phenotype, and the pathogen population structure, which provide vital insights into the epidemiology of gonorrhoea and AMR spread. However, linked epidemiological data were often limited; with gender and sexual orientation being the most commonly reported variables. Although details of sexual networks were inferred and the public health application of the study findings hypothesised, their value within public health interventions has yet to be systematically evaluated. Consequently, it remains unclear whether molecular epidemiological studies of *N. gonorrhoeae* are superior to traditional epidemiological studies without molecular data, such as routinely collected surveillance data and research studies using patient questionnaires of reported behaviour, when it comes to improving infection control or reducing the spread of *N. gonorrhoeae* and associated AMR.

Our review used systematic methods to identify and assess the relevant literature. The use of precise search terms applied to large medical literature databases reduced the risk of missing studies. Use of two independent reviewers to select studies and extract data minimised the risk of observer bias. A limitation of this review is that only English language studies were included.

The included studies highlight that molecular typing data are useful for understanding sexual networks, but also that there is often more than one explanation for the findings, which might make it difficult to determine appropriate public health actions. For example, a particular strain of *N. gonorrhoeae* identified in both MSM and heterosexuals might be explained by bridging between these sexual networks, or de novo independent generation of the sequence type. Similarly, where no sexual networks of sub-populations were identified, such as within the MSM community, this might be interpreted as being driven by disassortative sexual mixing and indicative of high rates of partner change, or it might be that there was insufficient epidemiological information collected to distinguish separate sexual networks.

We found it important to understand the sampling strategy used by molecular epidemiological studies to interpret the results and application within public health interventions. There is a risk of low internal and external validity if authors use restrictive sampling criteria, such as a specific AMR phenotype, or use a convenience sample without consideration of the isolates which are excluded. Inappropriate extrapolation is compounded if studies compare findings or use data from other studies without consideration of the different sampling strategies used. In order to improve the validity and use of molecular epidemiological studies, the sampling strategy should aim to reduce the chance of selection bias, for example by including consecutive isolates across a representative sample of the population. However, we note that the aim and objectives of the studies identified in this review varied and were not always intended to identify sexual networks.

We found conflicting results with respect to associations identified between particular patient variables and sequence types, such as whether different strains of *N. gonorrhoeae* are circulating amongst people living with HIV compared to those not living with HIV. These differences are likely due to the different populations and sexual behaviours of people in the areas under investigation. Given that populations and their behaviours may differ markedly, care should be taken when extrapolating findings from one area to another, and local analyses may be required. Consequently, the public health action from these findings may also be specific to local areas.

Many of the studies have hypothesised how molecular data might support the development and evaluation of public health interventions. Some studies demonstrated how molecular data were used to confirm clustering of infections in an outbreak. However, many of the other suggested public health applications, such as tailoring and targeting health promotion messages to specific groups or by changing clinical management protocols (for example by recalling patients back for test-of-cure depending on the infecting strain of the molecular data) are yet to be applied or properly evaluated.

In conclusion, linking DNA-based sequence typing data to patient-level epidemiological data can improve scientific knowledge on the structure of sexual networks and dissemination of *N. gonorrhoeae* and associated AMR. Future molecular epidemiological studies should, however, place greater emphasis on determining specific applications of molecular typing within public health interventions for controlling gonorrhoea. Greater collaboration between those involved in molecular epidemiological research and public health practitioners might facilitate evaluation of the public health potential of these increasingly prevalent technologies.

## Author contribution

KT, HB, MC, SC, NF and GH developed the protocol for the study. KT, HB and SC conducted the literature review and data extraction. All authors contributed to the writing of the manuscript.

## Appendix Supplementary materials

Image, application 1
