# Supplementary material for: Neisseria gonorrhoeae molecular typing for understanding sexual networks and antimicrobial resistance transmission: A systematic review
Source: J Infect. 2018 Jun;76(6):507–14. doi: 10.1016/j.jinf.2018.02.011 (PMC5999358; doi:10.1016/j.jinf.2018.02.011)
Supplement: Supplementary file 1 [file mmc1.docx]

**Supplementary Web Material**

**Systematic review search strategy**

For each search concept, subject headings and keywords were identified. A separate search was conducted for each subject heading and each keyword group, and the results combined to form the database result for each search concept. Each search concept was combined with *Neisseria gonorrhoeae* to restrict the search to studies related to this pathogen only.

| **Search concept** | **Subject heading** | **Keyword group** |
| --- | --- | --- |
| *Neisseria gonorrhoeae* | “neisseria gonorrhoeae” | “neisseria gonorrhoeae” or “n gonorrhoeae” or “gonococcus neisseria” or “micrococcus gonorrhoeae” or “neisseria gonococcus” or “neisseria gonorrhoea” or “neisseria gonorrheae” |
|  | gonorrhea | gonorrhea or “gonococcal infection” or “gonococcal infections” or gonococcosis or “gonococcus infection” or “gonorrhea epidemiology” or “gonorrhea treatment” or gonorrhoea or “neisseria gonorrhoeae infection” |
|  | “gonococcal arthritis” | “gonococcal arthritis” or “gonococcal septic arthritis” or “gonorrheal arthritis” |
|  | “gonococcal conjunctivitis” | “gonococcal conjunctivitis” or “gonococcal opthalmia” or “gonorrheal conjunctivitis” or “gonorrheal opthalmia” or “neisseria gonorrhoeae conjunctivitis” |
|  | “gonococcal urethritis” | “gonococcal urethritis” or “gonococal urethritis” or “gonococcic urethritis” or “gonococcus urethritis” or “gonorrheal urethritis” or “gonorrhoic urethritis” or “specific urethritis” or “urethritis gonorrhoica” |
|  |  | "gonococcal strain" |
|  |  | "gonococcal strains" |
| *Neisseria gonorrhoeae* multiantigen sequence typing (NG-MAST) | *no subject headings* | “neisseria gonorrhoeae multiantigen sequence typing” |
|  |  | “neisseria gonorrhoeae multi antigen sequence typing” |
|  |  | “neisseria gonorrhoea multi antigen sequence typing” |
|  |  | “neisseria gonorrhoea multiantigen sequence typing” |
|  |  | “gonorrhoea multiantigen sequence typing” |
|  |  | “gonorrhea multiantigen sequence typing” |
|  |  | “NG-MAST” |
|  |  | “NGMAST” |
|  |  | “neisseria gonorrhoeae MAST” |
|  |  | “gonorrhoea MAST” |
|  |  | “gonorrhea MAST” |
|  |  | “multiantigen sequence typing” |
|  |  | “multi antigen sequence typing” |
|  |  | “multi antigen sequence type” |
|  |  | “multiantigen sequence” |
|  |  | “multi antigen sequence” |
| Multilocus sequence typing (MLST) | “multilocus sequence typing” | “multilocus sequence typing” or “multilocus sequence analysis” |
|  |  | "multi locus sequence typing” |
|  |  | “multilocus sequence data” |
|  |  | “multi locus sequence data” |
|  |  | “multilocus sequencing” |
|  |  | “multi locus sequencing” |
|  |  | “multilocus typing” |
|  |  | “multi locus typing” |
|  |  | “multilocus type” |
|  |  | “multilocus typing” |
| Genome sequencing | "Genome" | “genome” or “genome components” or “genome, protozoan” or “protozoan genome” |
|  | "bacterial genome" | "bacteria genome" or "bacterium genome" or "genome, bacterial" |
|  | "microbial genome" | "microbial genome" or "prokaryote genome" or "prokaryotic genome" |
|  | "genome analysis" | “genome analysis” or “genome organisation” or “genome organization” |
|  | "gene library" | “gene library” or “genome library” or “genomic library” or “library, gene” |
|  | "metagenome" | "metagenome" or "metagenomes" or "metagenomic" or "metagenomics" |
|  | "genomics" | "genomic" or “genomics” or “biomics” |
|  | “genome DNA” | “genome DNA” or “DNA, genome” or “DNA, genomic” |
|  | "phylogenomics" | "phylogenomics" or "phylogenomic" |
|  | “gene mapping” | “gene mapping” or “gene map” or “genetic map” or “genetic mapping” or “genome mapping” or “genomic mapping” or “map, gene” or “mapping, gene” or “mapping, genetic” or “mapping, genome” |
|  |  | "whole genome sequencing" or "whole genome sequence" or "whole genome sequences" or "whole genomic sequence" or "whole genomic sequencing" or "genome sequencing" or "genome sequence" or "genome sequences" or "genomic sequencing" or "genomic sequence" or "genomic sequences" |
| Molecular typing | "molecular typing" | "molecular typing" |
|  |  | "genetic typing" or "gene typing" or "genome typing" or "genomic typing" or "genomes typing" |
| Phylogenetics | "molecular phylogeny" | "molecular phylogeny" or "genetic relationship" or "relationship,genetic" |
|  | "phylogeny" | "genealogical relationship" or "phylogenesis" or "phylogenesis model" or "phylogenesis analysis" or "phylogenetic analysis" or "phylogenetic relationship" or "phylogenetics" or "phylogeny model" or "phylogeny relationship" or "relationship, genealogical" or "relationship, phylogenetic" or "phylogenetic" or "phylogeny" |
|  | "phylogenetic tree" | "phylogenetic tree" or "dendrogram" or "evolution tree" or "evolutionary tree" or "phylogeny tree" |
| Molecular epidemiology | "molecular epidemiology" | "molecular epidemiology" or "epidemiology, molecular" |
| Genotype | "genotype" | "genotype" or "genotyping" |

**Conference abstract search**

Abstract books for the following conferences were searched for relevant studies: World STI & HIV Congress (2013 & 2015), International Union against STIs (IUSTI) Europe (2014 & 2015), IUSTI Asia-Pacific (2014), British Association of Sexual Health and HIV (BASHH) (2014 & 2015), STD Prevention conference (2014), European Congress of Clinical Microbiology and Infectious Diseases (ECCMID) (2014 & 2015), Interscience Conference on Antimicrobial Agents and Chemotherapy (ICAAC) (2014 & 2015) and the Federation of European Microbiological Societies (FEMS) (2013 & 2015).


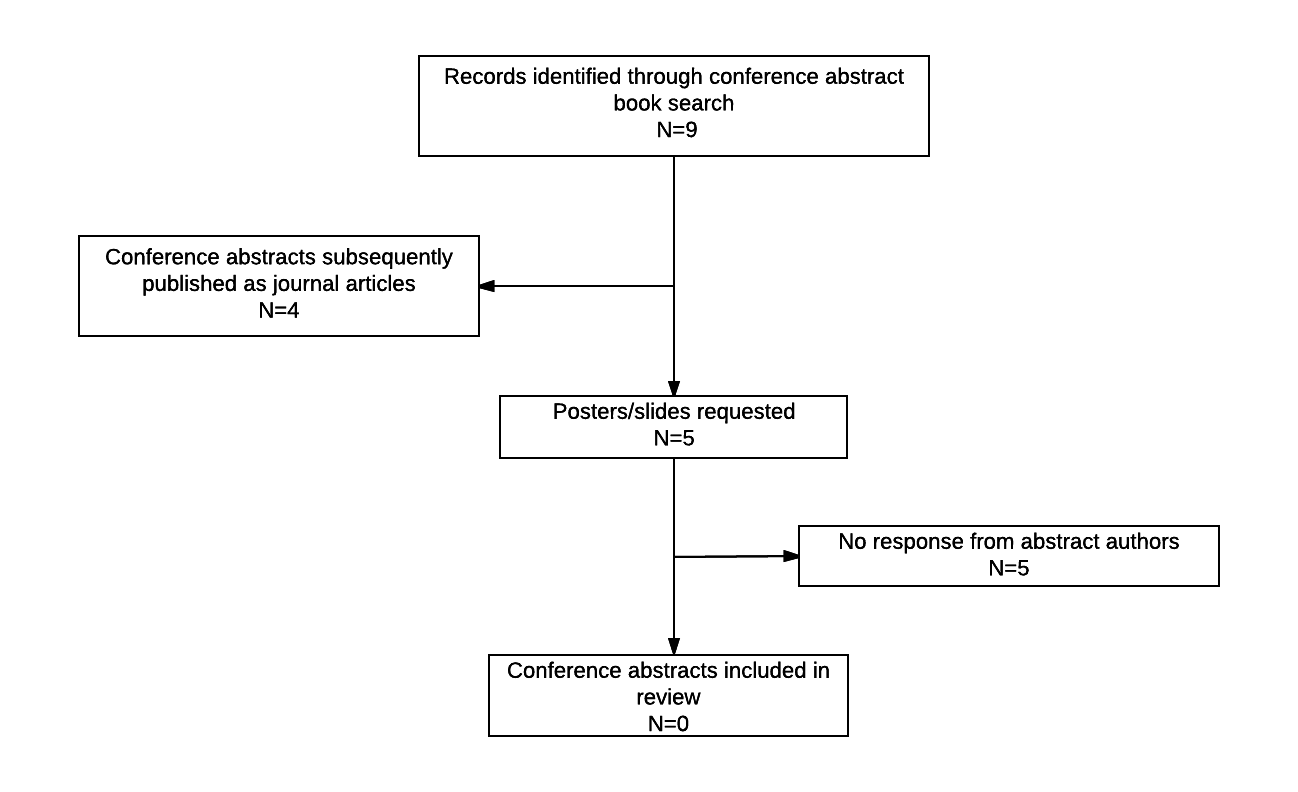
PRISMA flowchart indicating the systematic selection of conference abstracts for inclusion in this review

**Glossary of terms**

| Antimicrobial resistance (AMR) | The ability of a microorganism to grow in the presence of a concentration of an antimicrobial that would otherwise have killed or prevent growth of the organism |
| --- | --- |
| Cluster | In reference to molecular epidemiology, a cluster is a group of isolates that are similar according to their molecular data |
| Decreased susceptibility (DS) | An MIC value that demonstrates a microorganism is less sensitive to an antimicrobial but not resistant |
| Deoxyribonucleic acid (DNA) | Sequence of nucleotides present within organisms |
| Gene | A distinct sequence of nucleotides that code for a protein |
| Genome | All of the genetic material within an organism |
| Isolate | A culture of a microorganism |
| Minimum inhibitory concentration (MIC) | The concentration of antimicrobial at which the bacterium can no longer grow |
| Molecular epidemiology | The use of molecular typing methods for infectious agents in the study of the distribution, dynamics, and determinants of health and disease in human populations |
| Multi-Locus Sequence Testing (MLST) | Molecular typing technique used for *N. gonorrhoeae*: the nucleotide sequence of the internal fragments of seven or more chromosomal housekeeping genes are identified and used to categorise isolates into strain types |
| *Neisseria gonorrhoeae* Multi-Antigen Sequence Testing (NG-MAST) | Molecular typing technique used for *N. gonorrhoeae:* the nucleotide sequence of the porin PorB gene (porB) and the subunit B of the transferrin-binding protein gene (tbpB) are identified and used to categorise isolates into strain types |
| Nucleotide | The basic structure of nucleic acids, such as DNA |
| Sequence type | A genetic variant or subtype of a microorganism defined by the nucleotide sequence of the loci in the scheme used for molecular typing, such as the MLST sequence type or NG-MAST sequence type |
| Surveillance | In the context of healthcare, surveillance is the collection, analysis and interpretation of health-related data used to monitor the trends and design interventions for public health |
| Whole genome sequencing (WGS) | The process of identifying all the nucleotides in the DNA of an organism |

**Summary of included studies**

NG = *Neisseria gonorrhoeae*

*Variables relating to NG molecular typing data and patient epidemiological data or antimicrobial resistance (AMR): 1=sexual orientation or gender, 2=age, 3=location, 4=site of infection, 5=HIV status, 6=travel, 7=ethnicity, 8=symptoms, 9=other sexual behaviour, 10=AMR, 11 = STI history/co-infection

| **Reference** | **Aim** | **Time period** | **Setting & location** | **Sample size typed** | **Sample selection criteria for typing** | **Study conclusion summary** | **Variables linked between molecular & patient data*** |
| --- | --- | --- | --- | --- | --- | --- | --- |
| ***Studies using NG-MAST*** | | | | | | | |
| Martin (2004)^1^ | Demonstrate the method and application of NG-MAST to identify of individuals within a community who recently have been infected by the same gonococcal strain | Jun-Aug 2000 | 13 STI clinics in London, UK | 464 | Selected diverse strains (18); sexual contact pairs (10); alternate sampling of surveillance programme isolates (195); all resistant isolates from surveillance sample (241) | New approach to typing can identify linked cases of NG; different sized sequence type clusters indicate local transmission or repeated importation; large clusters in cities are indicative of an outbreak and can identify people for interventions | 1, 3, 6, 10 |
| Martin (2005)^2^ | Analyse the transmission of ciprofloxacin resistant NG by combining genotypic information with demographic and behavioural characteristics of the patients infected | 2000-2003 | 7 STI clinics in London, UK | 192 | Ciprofloxacin resistant isolates only | In earlier years ciprofloxacin resistance predominated in heterosexuals who had sex abroad but in later years was more likely to be found in MSM populations. Sequence type clusters increased in size as there was less infection associated with sex abroad and more ciprofloxacin resistance identified in MSM | 1, 6, 9, 10, 11 |
| Palmer (2005)^3^ | Compare the discrimination of auxotype/serovar typing with NG-MAST using NG isolates exhibiting reduced susceptibility or resistance to ciprofloxacin, and describe the epidemiology of these isolates | 2002 | Isolates referred to the referred to National Reference Laboratory from Edinburgh, Glasgow, Aberdeen, Scotland, UK | 106 | Isolates with reduced susceptibility or resistance to ciprofloxacin | NG-MAST provided more detail to the epidemiological data regarding clusters of infection; sequence types showed multiple transmission networks exist; sequence type clusters were transmitted exclusively among separate sexual networks but sequence type also found within the same networks; unique sequence type associated with travel suggesting importation | 1, 3, 6, 10 |
| Palmer (2006)^4^ | Investigate ciprofloxacin-resistant isolates with serogroup WI to determine whether one or more strain types accounted for the increase in these isolates | 2003 | Scotland, UK | 56 | Only ciprofloxacin-resistant serogroup WI isolates were typed and were from a previous study | Most cases were from MSM patients who acquired their gonococcal infections within the UK. Onward transmission of this sequence type facilitated by dense sexual networks as patients reported multiple sexual partners. Bridging to heterosexual populations may have occurred as some patients reported as bisexual, but partner tracing did not substantiate this | 1, 6, 9 |
| Lundback (2006)^5^ | Phenotypically and genetically characterise Swedish azithromycin-resistant NG isolates in order to examine the genetic homogeneity/heterogeneity of azithromycin resistant *NG,* and investigate the transmission of one specific azithromycin-resistant strain in the Swedish community | 1999, 2002, 2003 and 2004 | Stockholm, Sweden | 14 | Azithromycin resistant isolates only (1999 n=1, 2002 n=1, 2003 n=2, 2004 n=10) | All high-level azithromycin resistant isolates were the same sequence type; this sequence type is domestically transmitted; possibly also linked to importation | 9, 10 |
| Choudhury (2006)^6^ | Identify clusters of linked patients using typing data in the absence of contact tracing data | June-Nov 2004 | 13 STI clinics in London, UK | 2,045 | Any isolate from patients attending STI clinics selected, these are part of the national sentinel surveillance programme | Clusters of patients infected with the same strain showed similarities in behavioural and demographic features, which authors conclude provides evidence that these individuals are part of the same sexual network. Several large strain clusters were identified within MSM and 14 large strain clusters identified in heterosexuals. Authors concluded these strains represent the major endemic strains circulating within networks of MSM and heterosexuals. Other strains that were only identified once authors conclude were probably due to importation but could also be due to missed infections in the sampling. However, heterosexuals with unique strains had a higher proportion of individuals that had sex outside of the UK, suggesting importation of novel strains to London. The association between novel sequence type and sex abroad was not found in MSM. These major strains are circulating in lots of different ethnic groups. Bisexual men usually have strains similar to MSM so suggest gonococcal infection is spreading between men more than between men and women | 1, 2, 5, 6, 7, 8 |
| Bilek (2007)^7^ | Examine the concordance between the genotypes of isolates of NG from recent sexual contacts | Mar 1995-Dec 1998 and May 1999-Apr 2000 | STI clinic, Sheffield, UK | 1995-98 dataset: 112 (56 pairs of sexual contacts) 1999-2000 dataset: 119 | Known sexual contacts identified during time period | Concordance between sequence types of known sexual contacts was high, authors indicate that this means NG-MAST is a suitable tool as an adjunct to contact tracing for reconstructing sexual networks | 9 |
| Unemo (2007)^8^ | Investigate whether one strain of NG is responsible for the widespread transmission of ciprofloxacin-resistant infection, particularly among young heterosexuals | 2002-2003 | Gavleborg County and Cities in the Northern and Southern parts of Sweden | 47 | Isolates selected based on serotype (IB-10 only) | Confirmation of domestic spread of one ciprofloxacin-resistant NG strain in Sweden in young heterosexuals | 1, 10 |
| Risley (2007)^9^ | Explore the geographical distribution of NG across London, sexual orientation networks, ethnicity and social deprivation, and assess if there is local transmission according to molecular typing and geographical clustering of cases | Jun-Nov 2004 | 13 STI clinics in London, UK | 1,882 | Any isolate from patients attending STI clinics selected, these are part of the national sentinel surveillance programme | Sequence types clustered more in heterosexual men and women than in MSM, the authors suggest that MSM may be less likely to acquire infections locally | 1, 2, 3, 7 |
| Palmer (2008)^10^ | investigate the characteristics of NG isolates with decreased susceptibility to azithromycin and associated patient demographics | Apr 2004-Dec 2007 | STI clinics, Scotland, UK | 3,326 | Any isolate from patients attending STI clinics selected | Most sequence types contained a mix of azithromycin resistant and susceptible isolates and there was no demographic separation between these patients. However, the distribution of sequence types over time suggests that the development of resistance to azithromycin may have occurred several times within these different sequence types within discrete sexual networks | 1, 2, 3, 10 |
| Wong (2008)^11^ | Assess the genotype distribution of NG in Taiwan amongst patients to identify core groups at high risk of infection and investigate the domestic and international transmission of antibiotic resistant infections in specific sexual networks | Apr 2006-Aug 2007 | STI clinic, Taipei Hospital, Taiwan | 149 | Consecutive diagnoses made in the clinic | The large sequence type clusters suggested that multiple clonal transmissions existed in Taiwan. Some sequence types only identified within specific sub-groups of the populations such as HIV-positive MSM patients and these sequence types are where most of some AMR isolates are. For example, ST457 is only found in HIV-positive MSM and this ST457 makes up 65% of ciprofloxacin resistant isolates. Therefore the authors report that it is probably ST457 was introduced into Taiwan through MSM | 1, 5, 10 |
| Starnino (2008)^12^ | Assess: (i) the epidemiological characteristics of patients with gonorrhoea and the rate of HIV-1 co-infection; (ii) the serovar of NG isolates and the antimicrobial susceptibility to five antimicrobial drugs; and (iii) the correlation between molecular types and specific antimicrobial profile | Apr 2003-Jun 2005 | Seven STI clinics in Italy: Turin x2, Milan, Brescia and Bologna, Rome and Bari | 164 | 50% of isolates from consecutive sample from sentinel surveillance which had sexual orientation data | HIV status not associated with AMR; large sequence type cluster associated with heterosexuals; clonal dissemination of AMR through one sequence type likely; high number of different sequence type found in only one isolate | 1, 3 |
| Abu-Rajab (2009)^13^ | Assess the strain type similarities between isolates from sexual contacts and the association between strain types and patient demographic and behavioural data | Feb 2003-Jan 2004 | STI clinic, Glasgow, Scotland, UK | 170 | Consecutive isolates | Sequence type associated with sexual orientation but no other epidemiological variables that might be indicative of more specific sexual networks such as geographical clustering | 1, 2, 6, 9, 11 |
| Starnino (2009)^14^ | Describe the prevalence of azithromycin resistant NG and characteristics of infected patients | Jan 2007-Jun 2008 | 4 STI clinics in four cities (Milan, Turin, Rome, Bologna), Italy | 22 | Only azithromycin resistant isolates from male patients attending STI clinic selected | Some clustering of specific sequence types by residence, sexual orientation or AMR | 1, 10 |
| Fernando (2009)^15^ | Describe the characteristics of patient s infected with common NG strain types | Apr 2004-Mar 2006 | 1 STI clinic, Edinburgh, Scotland, UK | 370 | All culture-confirmed NG positive patients who attended the Edinburgh STI clinic | Patients with repeat gonococcal infections had different NG sequence types at repeat attendances. The authors suggest that this is evidence that these individuals were part of more than sexual network. Identical sequence types found in 94% of known sexual partners. Patients with unique sequence types significantly more likely to have had sex outside of the study area. Possible evidence of sexual networks that extend beyond the study area as the common sequence types were also reported in a London study previously. Distinct transmission networks were present in Lothian as unique sequence types found here. Some clustering of sequence types amongst different patient characteristics including gender, sexual orientation and HIV positivity | 1, 3, 5, 9 |
| Chisholm (2009)^16^ | Investigate the origin of high-level azithromycin resistance in NG in England and Wales | 2006-2007 | STI clinics, England, UK | 75 | Purposive sampling of azithromycin resistant and azithromycin sensitive isolates from sentinel surveillance programme plus in areas with high-level azithromycin resistance | High-level azithromycin resistance was identified in six closely related sequence types | 10 |
| Monfort (2009)^17^ | Analyse circulating NG to identify clusters of isolates from high-risk groups and clusters with particular antibiotic resistance phenotypes | Jan-Jun 2006 | Laboratories across France | 93 | 67 purposive selection of isolates with different AMR profiles, from different locations, from patients with different gender and different sites of infection. Additional 26 from rectal samples MSM to describe cluster within specific population | No geographic clustering of sequence types was observed and no predominant sequence types in MSM | 10 |
| Starnino (2010)^18^ | Identify the sequence types most represented among the ciprofloxacin-resistant NG isolates | 2003-2005 and 2007-2008 | Turin, Milan, Brescia, Bologna and Rome, Italy | 137 | Ciprofloxacin resistant isolates from patients that provided sexual orientation data | Exclusive transmission of sequence types within MSM or heterosexual networks | 1 |
| Florindo (2010)^19^ | Determine the NG antibiotic phenotype, genotype distribution and association with sexual orientation and age | 2004-2009 | Laboratories across Portugal | 236 | Over 100 local laboratories were asked to send NG isolates to national reference laboratory: 25 participated | Substantial differences were identified in sequence type distribution by age and sexual orientation. However, the authors state that as there was such a large diversity of sequence types, they could not identify specific sexual networks that spread NG. 8/9 sexual partners included in this study were infected by isolates with the same sequence type | 1, 2, 10 |
| Chisholm (2011)^20^ | Explore the characteristics and molecular epidemiology of NG isolates with decreased susceptibility to cefixime | 2005-2009 | STI clinics, England & Wales | 96 | Only typed cefixime decreased susceptibility isolates identified within national sentinel surveillance programme | Cefixime decreased susceptibility is clonally spread and circulating among MSM and heterosexual patients, some of whom reported sex abroad suggesting possible importation | 1, 6, 10 |
| Yuan (2011)^21^ | Describe baseline data from nationwide surveillance of NG susceptibility to azithromycin and genetically characterise azithromycin resistant strains | Jan 2008-Nov 2009 | Two STI clinics in Nanjing and Chongqing, China | 17 | Isolates exhibiting azithromycin resistance | NG-MAST provided additional information linking cases; different sequence type identified for azithromycin resistance than previous studies | 10 |
| Ota (2011)^22^ | Describe demographic characteristics and genotypic clustering of quinolone resistant NG | Nov-Dec 2006 | Isolates sent to reference laboratory, Ontario, Canada | 104 | Only ciprofloxacin resistant isolates typed | This study identified multiple QRNG clusters that were associated with specific sexual networks. There was also evidence of QRNG spread between MSM and non-MSM networks, as the same sequence types were identified in both populations | 1, 10 |
| Carannante (2012)^23^ | Present the circulating strain types and antimicrobial resistant profile of NG | 2003-2012 | Cases reported to Ministry of Health, Italy | 120 | Samples from patients that provided sexual orientation data from previous study (Starnino 2008), resistant isolates only | Common sequence type identified in MSM but also high variability in sequence type among MSM suggest lack of conditions favouring clonal spread of AMR or high recombination rate | 1 |
| Hjelmevoll (2012)^24^ | Investigate the genotypic and phenotypic properties (including antimicrobial resistance determination) of NG | 2009 | Six university hospitals in Norway: Tromsø, Trondheim,Stavanger, Bergen, Oslo x2 | 126 | Consecutive isolates | Sequence type circulating are heterogeneous; sequence type variety related to widespread importation of NG identified from epidemiological data | 1, 10 |
| Cole (2013)^25^ | Understand the factors affecting the transmission of NG in South East Wales, UK | May 2005- Sept 2006 | Three primary diagnostic laboratories covering STI clinics, GPs and other unspecified services in South East Wales | 475 | Consecutive isolates received by laboratories | Common sequence types widely disseminated in the UK in different sexual networks (MSM, heterosexuals); most localised clustering of sequence type in young heterosexuals; sequence type persistence over time within these networks demonstrates a lack of bridging between the networks | 1, 10 |
| Bernstein (2013)^26^ | Use molecular epidemiology to monitor the local epidemiology of NG | 2009 | San Francisco City STI Clinic, California, USA | 212 | Molecular typing data described for MSM only as small number of heterosexual men (no women), sample from national surveillance programme (first 25 cultured isolates every month) | Specific sociodemographic, behavioural, and phenotypic markers of antibiotic resistance clustered by sequence type. Among MSM, sequence type was significantly associated with ethnicity, number of sex partners, and reporting oral sex as the only recent urogenital exposure | 1, 7, 9, 10 |
| Chen (2013)^27^ | Describe a cluster of 47 NG cases belonging to NG-MAST ST4378 and MLST ST1901 | Apr 2006-Jun 2012 | Taipei City Hospital, Taiwan | 47 | Only isolates identified as ST4378 from surveillance programme | Sequence type along with AMR and geographical information confirmed isolates were in the same distinct high-risk MSM sexual network; this is different to existing data that suggested this clone was circulating in heterosexual networks; identified possible bridging between networks | 1, 10 |
| Ison (2013)^28^ | Describe changes in AMR and treatment options for NG | 2007-2011 | 26 STI clinics across England and Wales | 534 | Only cefixime decreased susceptible isolates typed from sentinel surveillance programme | Cefixime decreased susceptible isolates were associated with genotype 1407 clones, which were found predominantly in MSM | 1, 10 |
| Chisholm (2013)^29^ | Assess the public health benefit of NG-MAST molecular epidemiological typing of NG within Euro-GASP | 2010 | 21 EU/EEA countries, isolate sent to sentinel surveillance programme | 1,066 | Consecutive isolates from the European sentinel surveillance programme | Considerable diversity of gonococcal sequence types exist both within and between countries, some sequence types predominate. Some sequence types are associated with specific sexual networks, such as MSM, but these strains are also identified in heterosexual networks as well | 1, 3, 10 |
| Singh (2013)^30^ | Define populations of circulating NG populations, identify clusters of infection and investigate the role of particular strains in gonococcal transmission | 2007-2011 | Two STI clinics in Alberta, Canada | 238 | Isolates routinely submitted to reference laboratory; these are resistant to penicillin, tetracycline, ciprofloxacin and/or azithromycin; additional cefixime decreased susceptible isolates also selected | Significant diversity of sequence type; multiple importations of cefixime decreased susceptible NG; separate sequence type clusters identified among MSM and heterosexuals | 1, 10 |
| Horn (2014)^31^ | Investigate the epidemiology of AMR NG in Germany | Oct 2010-Dec 2011 | 23 laboratories across Germany | 213 | Consecutive isolates sent to the selected laboratories | A variety of different sequence types were identified. G1407 was significantly more common among males than females. Genogroups G25 and G387 less common in male groups | 1, 2, 3, 10 |
| Carannante (2014)^32^ | Determine the sequence type of multi-drug resistant NG | Apr 2003-Dec 2012 | 14 laboratories across Italy | 81 | Multi-drug resistant isolates identified by the network of participating laboratories | Genogroup G1407 is the main genogroup among the MDR gonococci in Italy and the majority of these were from MSM | 1, 10 |
| Jeverica (2014)^33^ | Phenotypically and genetically characterize NG isolates to elucidate the molecular epidemiology of the emergence and spread of NG with decreased susceptibility and resistance to extended spectrum cephalosporins | 2006-2012 | Primary diagnostic laboratories in Slovenia | 194 | Any available gonococcal isolate in Slovenia during time period | AMR trends due to spread of several discrete sequence types; specific associations between sequence type and sexual orientation identified indicate spread of sequence type within sexual networks | 1, 10 |
| Cheng (2014)^34^ | Describe longitudinal trends in NG antimicrobial resistance | Apr 2006-Aug 2013 | STI clinic, Taipei, Taiwan | 1,090 | Symptomatic patients at Taipei sexual health clinic diagnosed in that time period | Significant difference between sequence types circulating between heterosexual men and MSM. Change in the sequence types in MSM indicate different sources of sequence types | 1, 10 |
| Stevens (2015)^35^ | Describe high-level azithromycin resistant NG in Australia using national surveillance data | 2011-2013 | New South Wales, Victoria and Queensland, Australia | 6 | High-level azithromycin resistant isolates only | Five different sequence types with azithromycin resistant suggests independent generation of AMR; sequence type likely imported from China but also domestic transmission | 6 |
| Chen (2016)^36^ | Describe antimicrobial resistance, molecular epidemiology and genetic determinants of ceftriaxone resistance | 2012-2013 | STI clinic attendees, 11 sentinel sites, China | 920 | Selected isolates from clinics participating in the national sentinel surveillance programme for NG AMR | Sequence types of NG from China are different from other countries as several common genogroups have never been reported elsewhere. The authors state this indicates the domestic sexual networks are not strongly linked to sexual networks abroad. Authors also found that sequence types varied by geographic area within China | 10 |
| Foster (2016)^37^ | Describe the approaches for investigating and implementing control measures during an NG outbreak, including using molecular typing | 2010-2013 | North East England, UK | 284 | Isolates available from outbreak and non-outbreak region (n=169) plus isolates from sentinel surveillance in the outbreak area from previous years (n=115) | Different distribution of NGMAST sequence types identified between cases in outbreak compared to cases not in the outbreak leading to the conclusion that there was an association in outbreak cases between genogroup G25 and young heterosexuals | 1, 2 |
| Ni (2016)^38^ | Identify the prevalence of azithromycin resistance in NG | 2011-2012 | Hospital, Hangzhou, China | 118 | All NG cases with culture from one hospital | Azithromycin resistance prevalence was 21%; high-level azithromycin resistance prevalence was 18%. Majority of high-level azithromycin resistant cases were the same NG-MAST sequence type and were from men and women | 10 |
| Lahra (2017)^39^ | Describe an outbreak of azithromycin resistant NG in South Australia | Jan-Jun 2016 | South Australia | 28 | Isolates exhibiting azithromycin resistance from national antimicrobial resistance surveillance programme | Majority of isolates had the same NG-MAST sequence type (n=25) and all isolates from heterosexual patients. One patient was a sex worker and two had sex worker contacts but unclear if these patients had different NG-MAST sequence types to majority of sample | 10 |
| Serra-Pladevall (2017)^40^ | Describe NG antimicrobial resistance and strain type differences between MSM and heterosexual populations | Jan-Dec 2013 | STI clinic, Barcelona, Spain | 111 | All isolates from heterosexuals attending STI clinic, random selection of similar number from MSM attending clinic | Significant differences between the NG strains circulating amongst MSM and heterosexuals in terms of antimicrobial resistance and NG-MAST sequence type | 1, 10 |
| ***Studies using MLST*** | | | | | | | |
| Perez-Losada (2007)^41^ | Infer the evolutionary history of isolates to determine if genetically variant strains of NG diverged before or after quinolone resistant NG first appeared in Israel and estimate the selective pressure on each gene and examined the association of selected sites with the ciprofloxacin resistance phenotype | Jan 2000-Oct 2001 | Tel Aviv, Israel (clinic/lab not specified) | 48 | Random selection of resistant and susceptible quinolone isolates from previous study | Phylogenetic analyses suggested that quinolone resistant were imported into Israel on at least three separate occasions. Patients reporting sex with sex workers who are likely to harbour multiple gonococcal strains which would increase the chances for horizontal gene transfer | 9, 10 |
| Trembezki (2016)^42^ | Describe the genetics and distribution of gonococcal AMR in Australia | Jan-Jun 2012 | Laboratories participating in national surveillance, Australia | 2,218 | Consecutive culture isolates received by laboratories | The ratio of males to females differed noticeably between certain sequence types. Although a large numbers of different gonococcal strains may be circulating within a population at any given time, *NG* infections on a population level are dominated by a relatively small number of strains | 1, 3, 10 |
| ***Studies using WGS*** | | | | | | | |
| Grad (2014)^43^ | Describe the relatedness between NG isolates to reconstruct the likely spread of lineages through sexual networks according to geography and sexual orientation, and to identify likely emergence events of resistant phenotypes | 2009-2010 | STI clinics, USA | 236 | Cefixime resistant plus sensitive isolates matched according to location, date and sexual orientation. Isolates from national sentinel surveillance programme | Cefixime resistance spreads eastward from California, predominantly within MSM networks, with a small number of introductions into heterosexual networks | 1, 3, 10 |
| Demczuk (2015)^44^ | Describe the dissemination, relatedness, and emergence of NG isolates with elevated extended spectrum cephalosporin MICs | 1989 and 2013 | Ontario, British Columbia, Quebec, Saskatchewan, Nova Scotia, Alberta, New Brunswick and Manitoba, Canada | 169 | Convenience sample with aim to include isolates that were across time, ceftriaxone decreased susceptible, of different NG-MAST strain types and locations | NG is genetically heterogeneous and broadly distributed; association identified between different lineages and patient characteristics suggesting link of specific lineages to heterosexual populations | 1, 10 |
| Chisholm (2016)^45^ | Describe an outbreak of high-level azithromycin resistant NG | Jan 2014 – Mar 2015 | Leeds & London, England | 16 | Eight cases defined as outbreak cases (high-level azithromycin resistant NG in Leeds) (seven sequenced), eight control strains chosen to be from similar area with varying azithromycin resistant profiles | WGS and NG-MAST showed genomes of outbreak cases to be very similar to each other (≤1 nucleotide difference) and different to the control isolates. Outbreak cases 1 nucleotide different to sequence type associated with previous high level azithromycin resistant cases | 3, 10 |
| De Silva (2016)^46^ | Define the genetic diversity between samples related by transmission, and apply this information to detect local, regional, and international transmission of NG | Jan 2011-Mar 2015 | Brighton, England | 1,061 | Consecutive culture positive NG samples from attendees at sexual health clinic and primary care. Additional convenience sample of previously published WGS data for comparison. | Identified a method of determining the plausibility of direct or indirect transmission between two cases using WGS data. Within Brighton found sustained local transmission between cases often within 1-3 months apart, possible reflection of frequent partner changes involving many infected individuals. Comparison to other datasets found evidence of possible link to cases elsewhere in the UK in 18% of samples and 9% to cases in the US | 6 |
| Didelot (2016)^47^ | Investigate how WGS data can be used to track transmission of NG within a city | Sheffield:1995-2000  London June to Nov 2004 | Sheffield & London, England | Sheffield: 132  London: 105 | Sheffield: isolates from one sexual health clinic identified as NG-MAST strain type 12  London: isolates from across city sexual health clinics identified as NG-MAST strain type 225 | Used WGS data to estimate time between transmission events to support prediction of linkage between two cases; cases sampled more than 8 months apart are unlikely to be in the same transmission chain. Likely transmission links were significantly associated with shorter geographical distances in London and there were more links between HIV positive individuals than HIV negative individuals | 1, 2, 3, 5, 9 |
| Grad (2016)^48^ | Define the distribution of resistance markers to the three most clinically relevant classes of antimicrobials for treatment of NG | 2000-2013 | STI clinics, USA | 1,102 | Purposive selection of isolates based on antimicrobial susceptibility profile focused predominantly on isolates with reduced susceptibility to cefixime, ceftriaxone, azithromycin and quinolones; isolates from national sentinel surveillance programme | Cefixime and ceftriaxone reduced susceptibility has spread predominantly through clonal expansions and is highly although not exclusively associated with the mosaic *penA* XXXIV allele and its derivatives. Azithromycin reduced susceptibility arises through multiple mechanisms that are less clonal. Quinolone resistance is mainly clonal and has emerged several times. | 1, 3, 10 |
| Jaccobson (2016)^49^ | Describe the genomics of azithromycin resistance | 2009-2014 | 17 European countries | 75 | Purposive selection of azithromycin resistant NG (MIC >2mg/L) from sentinel surveillance programme | Identified clonal spread of *NG* strains accounted for the majority of the azithromycin resistance in Europe. Future molecular prediction of clinical resistant to azithromycin should focus on detection of A2059G and C2611T mutations in the 23S rRNA gene | 3, 10 |

**Summary of risk of bias assessment for included studies**

NG = *Neisseria gonorrhoeae*

| **Reference** | **Is the study generalizable to the study population?** | **How many eligible isolates were included?**  **How much missing patient epidemiological data was there?** |
| --- | --- | --- |
| *Studies using NG-MAST* | | |
| Martin (2004)^1^ | Subset of national sentinel surveillance that collects consecutive isolates during 3-month period from 13 London clinics (N=195). Other sample contained all isolates resistant to antibiotics specified and that had previously been used to evaluate typing methods (N=268) | All isolates eligible for typing typed. No information on completeness of epidemiological data presented |
| Martin (2005)^2^ | All quinolone resistant isolates over four year period selected from national sentinel surveillance programme. Represent 3.6% (192/5260) of NG collected in programme. Unclear how much of all NG this represents overall | All isolates eligible for typing included. Sexual orientation available for all isolates. Ethnicity missing for 21.3% (41/192). No information on missing concurrent STI |
| Palmer (2005)^3^ | Selected isolates that were reduced susceptible or resistant to ciprofloxacin representing 13% (106/818) of all isolates submitted in time period. This is all of Scotland as all isolates are submitted to reference laboratory | All isolates eligible were typed (n=106). Sexual orientation reported for 90.6% (96/106) of cases. Area of acquisition known for all isolates. |
| Palmer (2006)^4^ | Selected subset of all isolates in Scotland in one year that were of particular serotype and ciprofloxacin resistant. This represented 44% (56/126) of all ciprofloxacin resistant isolates and 7% (56/824) of all NG in Scotland in this year | All eligible isolates sequenced. Epidemiological data reported for 86% (42/56) of isolates |
| Lundback (2006)^5^ | Study aim is to characterise all azithromycin resistant NG in Sweden in 2004, all of these isolates selected for inclusion | All isolates eligible sequenced. All epidemiological data included reported |
| Choudhury (2006)^6^ | Subset of isolates from national sentinel surveillance that have come from London clinics covering 77% of all reported gonorrhoea in London during study period | 81.1% eligible isolates successfully typed (2345/2891). Epidemiological data available for 87% of isolates typed (2045/2345). 11.6% (238/2045) missing ethnicity. 11.2% (230/2045) missing concurrent STI status. 7.5% (154/2045) missing previous gonorrhoea information. 7.3% (150/20458) missing symptoms information. 42.3% (866/2045) missing HIV status information. Further breakdown by sexual orientation available in Table 1 of paper |
| Bilek (2007)^7^ | Isolates from sexual contact pairs in a selected clinic during one year (Sheffield's main GUM clinic) included in the sample for sequencing (167). Unclear how many samples this represents for the whole year. Study is about concurrency of sequence type between sexual contacts so generalisability to location or patient group less relevant. Study conducted exhaustive contact tracing of all NG diagnoses during the study time period | All sexual contact pairs identified sampled. Gender data only provided for subset of patients in the sample that represented non-concordant genotypes in figure 1 |
| Unemo (2007)^8^ | Selected isolates of particular serotype and ciprofloxacin resistant thought to be part of a core group (N=20) and other selected ciprofloxacin resistant isolated (N=26) from 10 cities. Two ciprofloxacin susceptible isolates with same serovar type also used and was found circulating at the same time. Unclear representativeness of all NG | One isolate unavailable for typing from core group so 95% (19/20) successfully typed. Epidemiological data available for all |
| Risley (2007)^9^ | All NG cases diagnosed in 13 London GUM clinics during five month period. These clinics identify 80% of NG in London | Of all eligible isolates, 70% successfully retrieved and typed (2045/2891). Postcode data reported for 65% (1871/2891). Other epidemiological data complete |
| Palmer (2008)^10^ | All isolates reported to reference laboratory during time period, which would be all NG in Scotland (N=3,326) | All eligible isolates sequenced. Epidemiological data only reported for common sequence types so unclear how much missing epidemiological data in total |
| Wong (2008)^11^ | Subset of male diagnoses from all diagnoses made in one clinic during time period (93%: 139/149). Unclear how many isolates in city the isolates represent | All eligible samples sequenced. Sexual orientation reported for 95% (132/139) of isolates |
| Starnino (2008)^12^ | Isolates from 7/12 STI clinics in large cities participating in sentinel surveillance of STIs in Italy. No information about how many clinics there are in Italy. All isolates diagnosed during study period eligible (N=514) but 50% of isolates with a culture and patient sexual orientation data were eligible typed NGMAST (164/514). Unclear what proportion of NG in Italy this represents | All eligible isolates typed. All epidemiological data complete |
| Abu-Rajab (2009)^13^ | All isolates from selected clinic (Glasgow's main GUM clinic) included in sample to be sequenced (178). 4.5% (8/178) unable to be sequenced because of inviable sample or not matched to clinical data. 59% (105/178) not included in sexual network analysis as questionnaire not completed. Information on the representativeness of the included 41% (65/178) not provided | 59% of eligible sample missing from sexual network analysis because no questionnaire data, sample not viable or sample not matched to clinical records. No information provided on these missing patients to assess if different from the patients included in the sexual network analysis. Unable to assess if this is missing systematically or at random |
| Starnino (2009)^14^ | Subset of azithromycin resistant isolates (n=22) selected from four large STI clinics that represent ~70% of all NG cases (n=219, estimated denominator =313). Therefore in total sample represent ~7% (22/313) of isolates in Italy | Sequenced all eligible isolates. No missing epidemiological data |
| Fernando (2009)^15^ | Over the study period, all 370 culture-positive NG diagnoses at the Edinburgh GUM clinic were eligible Estimated representativeness of all NG in Scotland: 20.5% ((370/2)/900). Estimated representativeness of all NG in Edinburgh: 90.2% ((370/2)/205). No information on number of isolates during study period with no culture | 100% eligible isolates typed. Most epidemiological variables complete except location of recent sexual contacts missing for 12.2% (45/370) |
| Chisholm (2009)^16^ | Subset of isolates from the national sentinel surveillance programme selected for typing from one year (N=75). Isolates selected as either resistant to azithromycin (n=34) (no information about how many this represents from one year) or from two clinics were high level azithromycin resistance has been identified (n=41) (unclear how many diagnoses form all cases in these clinics these represent) | All eligible isolates successfully typed. 98.7% (74/75) reported sexual orientation. Age only reported for subset of high-level azithromycin resistant isolates |

| Monfort (2009)^17^ | Aim of study is to describe NG circulating in France. Selected a subset of isolates from voluntary laboratory reporting surveillance reported during 6 month period (31.5%; 93/295). Within this 72% (67/93) selected based on AMR profile (mix of resistant and susceptible to variable antimicrobials), geographic organs, gender and site of infection. Remaining selected based on site of infection (rectal) and from MSM 23% (26/93) | All selected isolates successfully typed. Sexual orientation and site of infection known for all selected isolates. Age not reported for 6 isolates |
| --- | --- | --- |
| Starnino (2010)^18^ | Subset of larger sample, selecting isolates that were resistant to ciprofloxacin and patient sexual orientation data is available. Represented 56% (137/244) of ciprofloxacin resistant isolates and 25% (137/599) of all isolates in sample. Unsure if total samples (n=599) is a consecutive from laboratories/clinics | All isolates eligible for sequencing sequenced. No further missing data than the missing data for sexual orientation of ciprofloxacin resistant isolates 44% (107/244) |
| Florindo (2010)^19^ | 25/100 laboratories sent isolates over four years. Unclear representativeness compared to all NG circulating in Portugal. Most labs sending isolates from one city (Lisbon). Some years sampled more than others (only 17 in 2004) | 86% eligible samples successfully typed(236/274). Age reported for all. Gender reported for all. Sexual orientation missing for 39% (92/236) |
| Chisholm (2011)^20^ | Subset of isolates from sentinel surveillance or isolates referred to national reference laboratory were typed if exhibiting decreased susceptibility to cefixime (N=97). Representativeness of sentinel surveillance: 1.2% (55/4649). Unknown denominator of isolates selected from the reference laboratory sample | 99% of isolates eligible for typing successfully tested (96/97). Gender reported for 98% (94/96). Age reported for 100%. Sexual orientation only known for subset of sentinel surveillance sample (81%: 44/54). Ethnicity or Information about travel-associate sexual partnerships known for subset of sentinel surveillance sample (79.6%: 43/54) |
| Yuan (2011)^21^ | Selected azithromycin resistant isolates representing 5% (17/318) of NG in two cities during time period. Unclear how many this would be for whole country | All eligible isolates sequenced. All epidemiological data complete |
| Ota (2011)^22^ | Selected a subset of consecutive quinolone resistant isolates during two month time period in 2006, representing 15%: (104/695) of all quinolone resistant isolates. This represents 4.2% (104/2482) of all NG in province studied in 2006 | All eligible isolates were typed. Sexual orientation and age data available for all isolates. 3% of isolates missing residence information |
| Carannante (2012)^23^ | Isolates from 6/12 STI clinics participating in sentinel surveillance of STIs in Italy. No information about how many clinics there are in Italy. Six clinics selected as they are located in large Italian cities. NG-MAST performed on 19.3% (120/620) of all NG diagnoses during study period (5 years) based on isolates resistant to antimicrobials (unspecified which antimicrobial) and from patients with known sexual orientation known. Unclear what proportion of NG in Italy this represents | No information about epidemiological profile of all isolates collected during time period (N=620) |
| Hjelmevoll (2012)^24^ | All viable isolates diagnosed in one year for six hospitals in Norway. Study covers 42% (114/269) NG in Norway. Representative of all NG in Norway with regard to gender, sexual orientation and place of contracting infection | 126 eligible cases, 114 successfully typed (90.5%). Majority of patients reported sexual orientation 98% (112/114) |
| Cole (2013)^25^ | All isolates collected at three main laboratories in region included. Unclear what proportion of all diagnoses in this region this covers | 93% (475/511) of eligible isolates typed. Epidemiological data available for 99.2% (507/511) of isolates. Gender reported for all isolates. Additional epidemiological data available for GUM patients only (69.9%: 357/511) |
| Bernstein (2013)^26^ | Sample includes first 25 male urethral specimens from symptomatic patients attending one clinic each month for one year. Unclear on the representativeness of these samples for this clinic. Only analysed sexual network data from MSM sample as low number of samples from men who have sex with women (20%; 53/265) | All isolates eligible for typing were included. Missing epidemiological data is reported in paper table 1. Only variable with missing data is HIV status (4.3%) |
| Chen (2013)^27^ | Aim of study was to describe subset of NG diagnoses with specific NGMAST (N=47). Subset from surveillance programme that covers 40 clinics in Taiwan (N=2,357). Unclear how many clinics in Taiwan | All eligible isolates included. 70% of isolates included in study linked to medical records (33/47) for additional epidemiological information. For these isolates, no further missing data |
| Ison (2013)^28^ | Subset of isolates from sentinel surveillance programme that tests ~7-10% of all circulating NG. Isolates exhibiting resistance to cefixime selected for typing, 7.4% (547/7378) of isolates collected in the programme during study period eligible for typing | 97.8% (535/547) eligible isolates successfully typed. Sexual orientation reported for 92% (505/547) of eligible isolates. Ethnicity reported for 89% (490/547) of eligible isolates. Number of sexual partners in the UK reported for 89% (486/547) of eligible isolates. Travel associated sexual partnerships reported for 73% (399/547) of eligible isolates. Symptomatic information reported for 65% (357/547) of eligible isolates. Chlamydia status reported for 92% (501/547) of eligible isolates. HIV status reported for 85% (463/547) of eligible isolates |
| Chisholm (2013)^29^ | Subset of NG across Europe from sentinel surveillance programme that collects 55 isolates from consecutive patients twice annually to be representative of national distribution of cases and have AMR data (overall representation of all NG in Europe: 3.3%; 1066/32028) | All eligible isolates typed. 96.6% (1030/1066) reported age. 98.5% (1050/1066) reported gender. 55% (586/1066) reported sexual orientation |
| Singh (2013)^30^ | All culture cases in area sent to regional lab for testing. This is 26.4% (2250/8535) of all NG in area during 2011-2007. Of these, isolates with resistance to penicillin tetracycline, ciprofloxacin, cefixime decreased susceptibility and/or azithromycin were typed (n=238: 2.8% of all NG (238/8535) and 10.6% (235/2250 of all culture diagnoses). Majority of isolates in study from 2009-2011 (95.8%; 228/238) | Unclear for earlier years how many isolates eligible for NGMAST but not typed. Missing data not reported for samples typed |
| Horn (2014)^31^ | 23 laboratories reporting consecutive NG isolates. Unclear how representative of Germany the NG identified in these laboratories | Unclear if more isolates eligible than successfully typed. Age and gender reported for all isolates. Site of infection not reported for 18.8% of isolates (40/213) |
| Carannante (2014)^32^ | Typed isolates that were multi-drug resistant (MDR) from a subset of NG diagnoses (66.5%; 1777/2671) available in Italy over 14 year period (3.0%; 81/2671). Study aim was to investigate strain types of MDR so sample generalizable to MDR NG cases | All eligible isolates were typed. No missing epidemiological data for typed isolates |
| Jeverica (2014)^33^ | 74% of all NG cases in Slovenia selected during time period | Assume all NG cases eligible so only typed 74% (N=194). Age and site of infection known for all isolates. Sexual orientation reported for 89% (179/194) of cases |
| Cheng (2014)^34^ | Per year, 1500 NG cases in Taiwan; 500 in Taipei City. Study conducted isolates from one hospital over 7 years in Taipei city collected 1111 isolates, ~10% of all NG in Taiwan | Nearly all isolates eligible typed (98.1%; 1090/1111). 100% gender reported. 98.2% reported sexual orientation (1070/1090). 94.5% reported HIV status (1030/1090). 89% reported syphilis status (971/1090) |
| Stevens (2015)^35^ | Subset of isolates from national surveillance over three year period that exhibited high-level azithromycin resistance . Not representative of all NG cases | All isolates eligible for sequencing sequenced. Complete information on gender, age, site for all. No data on completeness of country of acquisition but missing data for one acknowledged |
| Chen (2016)^36^ | One isolate per patient from 11 sentinel sites across China selected. Sites selected as detect the majority of STIs in each respective geographical location. In China in 2013, 103,085 NG cases, so report covers 0.9% of circulating NG | 73.2% of eligible isolates typed (920/1257). Epidemiological data available for of eligible isolates (N=1,257), unclear which isolates typed had missing epidemiological data. 99.7% (1254/1257) reported gender. 92.3% (1168/1257) reported sexual orientation. 94.3% (1160/1257) reported location of infection or whether they had sex abroad. 28.4% (357/1257) reported previous STI. 30.9% (388/1257) reported drug use history or previous use of antibiotics for genital infection |
| Foster (2016)^37^ | Isolates from an outbreak and controls from the same area and time were period were eligible for typing (N=623). Additional historical sentinel surveillance samples also selected (N=123) | Of the eligible samples, 27% (169/623) of the outbreak/non-outbreak cases were successfully typed and matched to epidemiological data for analysis. 93% (115/123) of the historical sample sequenced |
| Ni (2016)^38^ | All samples from the two years period from one hospital in China (n=118) | All eligible samples typed. Gender and age only reported for high-level azithromycin resistant cases. Unclear if epidemiological data missing for others |
| Lahra (2017)^39^ | Selected all azithromycin resistant isolates collected as part of sentinel surveillance. Unclear denominator for all NG cases in this time period | All isolates eligible sequenced. Epidemiological data missing for 3.5% (1/28) |
| Serra-Pladevall (2017)^40^ | All samples from heterosexuals and random selection of MSM attending one clinic (only STI clinic in city) in Spain in one year (N=107). Representative of 7.4% (53/715) MSM and 8.9% (54/606) of heterosexual men and women | All eligible samples sequenced. Gender, age and sexual orientation complete for all. Ethnicity missing for 9.4% (5/53) MSM, 27.8% (15/54) heterosexuals. Education status missing for 32.1% (17/53) MSM, 4.3% (25/54) heterosexuals. HIV status missing for 15.1% (8/53) MSM, 35.2% (19/54) heterosexuals. Sexual behaviour missing for 3.2% (6/53) MSM, 38.9% (21/54) heterosexuals |
| *Studies using MLST* | | |
| Perez-Losada (2007)^41^ | Selected subset of samples (N=48) half resistant to fluoroquinolones and half sensitive to fluoroquinolones from previous genotype study (60%; 48/80). This previous study was a random sample of male urethral isolates. This represents 9% (48/525) of all NG in Tel Aviv: the area of analysis | All isolates selected were successfully typed. All epidemiological data included complete |
| Trembezki (2016)^42^ | Isolates from surveillance programme representing 34.4% of all isolates (2452/7128) and 98% of all isolates cultured. Surveillance programme covers 30% of all gonorrhoea isolates in Australia | 90.4% of eligible isolates sequenced (2218/2452). Missing data for gender low: only 15 unknown. |
| *Studies using WGS* | | |
| Grad (2014)^43^ | Purposive sampling of cefixime resistant and susceptible isolates matched on location, date of isolation and sexual orientation. One year of study from sentinel surveillance programme, which selects first 25 male urethral samples from participating clinics each month. Cefixime resistant isolates represented 97% of all cefixime resistant isolates identified by surveillance programme in this time period. Susceptible isolates likely under represent all circulating NG in USA | Eligible isolates successfully sequenced (N=236). All epidemiological data complete |
| Demczuk (2015)^44^ | Isolates selected based on decreased susceptibility to ceftriaxone in archives spanning ~15 years (N=65). Isolates chosen are specific subset from different years to provide broad range of geographical distribution and antimicrobial susceptibilities (N=105). Unclear how representative of all NG during time period | All selected isolates for sequencing successfully sequenced. Gender reported for 99% of samples (168/169). Age reported for 81% (137/169) of isolates |
| Chisholm (2016)^45^ | Selected all isolates considered part of outbreak (epidemiologically linked cases that exhibit high-level azithromycin resistance) (N=7)) plus additional cases (N=6) with range of antimicrobial susceptibility data from same time period as controls but unclear how representative these controls are | All eligible isolates sequenced. Epidemiological data provided for the outbreak cases only |
| De Silva (2016)^46^ | Main sample included all consecutive culture NG samples from STI clinic in one city over four year period | 98% eligible isolates successfully sequenced (1407/1437). Gender missing for 2 patients. Sexual orientation only available for 15 pairs of cases. |
| Didelot (2016)^47^ | Two datasets (London and Sheffield), both restricted to isolates with most common ST therefore not representative of all NG circulating. This represents 3.4% of NG in London (127/3754) but in Sheffield, unclear how much sequenced isolates represent of all gonorrhoea circulating (n=140) | Missing eligible sample in London 15% (19/127), in Sheffield (8/140). Linked partner data available for Sheffield dataset but limited epidemiological data. London dataset reported if HIV status missing (31%; 33/105) |
| Grad (2016)^48^ | Sample collected based on antimicrobial susceptibility profile. Isolates from 14 years (N=1102) of sentinel surveillance programme, which collects first 25 male urethral isolates from participating clinics across USA each month. Unclear representativeness of all NG circulating | All eligible isolates sequenced. All have sexual orientation data |
| Jaccobson (2016)^49^ | Selected subset of international sentinel surveillance which collects 55 consecutive isolates during two time periods annually from 17 countries. All azithromycin resistant isolates from this surveillance programme within four year period selected (N=66) and nine additional isolates between 2013 and 2014 | All eligible isolates successfully typed. Gender missing for 4% (3/75). Sexual orientation missing for 47% (30/64) of male cases. Site of infection missing for 4% (3/75) |

**Reference list for Summary of Included Studies Table & Risk of Bias Table**

1. Martin IMC, Ison CA, Aanensen DM, Fenton KA, Spratt BG. Rapid sequence-based identification of gonococcal transmission clusters in a large metropolitan area. *Journal of Infectious Diseases* 2004; **189**(8): 1497-505.

2. Martin IMC, Ison CA, Aanensen DM, Fenton KA, Spratt BG. Changing epidemiologic profile of quinolone-resistant Neisseria gonorrhoeae in London. *Journal of Infectious Diseases* 2005; **192**(7): 1191-5.

3. Palmer HM, Young H, Martin IMC, Ison CA, Spratt BG. The epidemiology of ciprofloxacin resistant isolates of Neisseria gonorrhoeae in Scotland 2002: A comparison of phenolypic and genotypic analysis. *Sexually Transmitted Infections* 2005; **81**(5): 403-7.

4. Palmer HM, Young H. Dramatic increase in a single genotype of TRNG ciprofloxacin-resistant Neisseria gonorrhoeae isolates in men who have sex with men. *International Journal of STD and AIDS* 2006; **17**(4): 254-6.

5. Lundback D, Fredlund H, Berglund T, Wretlind B, Unemo M. Molecular epidemiology of Neisseria gonorrhoeae- identification of the first presumed Swedish transmission chain of an azithromycin-resistant strain. *Apmis* 2006; **114**(1): 67-71.

6. Choudhury B, Risley CL, Ghani AC, et al. Identification of individuals with gonorrhoea within sexual networks: a population-based study. *Lancet* 2006; **368**(9530): 139-46.

7. Bilek N, Martin IM, Bell G, Kinghorn GR, Ison CA, Spratt BG. Concordance between Neisseria gonorrhoeae genotypes recovered from known sexual contacts. *Journal of Clinical Microbiology* 2007; **45**(11): 3564-7.

8. Unemo M, Sjostrand A, Akhras M, et al. Molecular characterization of Neisseria gonorrhoeae identifies transmission and resistance of one ciprofloxacin-resistant strain. *Apmis* 2007; **115**(3): 231-40.

9. Risley C, Ward H, Choudhury B, et al. Geographical and demographic clustering of gonorrhoea in London. *Sexually Transmitted Infections* 2007; **83**: 481-87.

10. Palmer HM, Young H, Graham C, Dave J. Prediction of antibiotic resistance using Neisseria gonorrhoeae multi-antigen sequence typing. *Sex Transm Infect* 2008; **84**(4): 280-4.

11. Wong WW, Huang CT, Li LH, Chiang CC, Chen BD, Li SY. Molecular epidemiological identification of Neisseria gonorrhoeae clonal clusters with distinct susceptibility profiles associated with specific groups at high risk of contracting human immunodeficiency virus and syphilis. *Journal of Clinical Microbiology* 2008; **46**(12): 3931-4.

12. Starnino S, Suligoi B, Regine V, et al. Phenotypic and genotypic characterization of Neisseria gonorrhoeae in parts of Italy: detection of a multiresistant cluster circulating in a heterosexual network. *Clinical Microbiology & Infection* 2008; **14**(10): 949-54.

13. Abu-Rajab K, Palmer HM, Scoular A, et al. To what extent does Neisseria gonorrhoeae multiantigen sequence typing of gonococcal isolates support information derived from patient interviews? *International Journal of STD and AIDS* 2009; **20**(6): 414-7.

14. Starnino S, Stefanelli P. Azithromycin-resistant Neisseria gonorrhoeae strains recently isolated in Italy. *Journal of Antimicrobial Chemotherapy* 2009; **63**(6): 1200-4.

15. Fernando I, Palmer HM, Young H. Characteristics of patients infected with common Neisseria gonorrhoeae NG-MAST sequence type strains presenting at the Edinburgh genitourinary medicine clinic. *Sexually Transmitted Infections* 2009; **85**(6): 443-6.

16. Chisholm SA, Neal TJ, Alawattegama AB, Birley HD, Howe RA, Ison CA. Emergence of high-level azithromycin resistance in Neisseria gonorrhoeae in England and Wales. *Journal of Antimicrobial Chemotherapy* 2009; **64**(2): 353-8.

17. Monfort L, Caro V, Devaux Z, Delannoy AS, Brisse S, Sednaoui P. First Neisseria gonorrhoeae genotyping analysis in France: Identification of a strain cluster with reduced susceptibility to ceftriaxone. *Journal of Clinical Microbiology* 2009; **47**(11): 3540-5.

18. Starnino S, Dal Conte I, Matteelli A, et al. Trend of ciprofloxacin resistance in Neisseria gonorrhoeae strains isolated in Italy and analysis of the molecular determinants. *Diagnostic microbiology and infectious disease* 2010; **67**(4): 350-4.

19. Florindo C, Pereira R, Boura M, et al. Genotypes and antimicrobial-resistant phenotypes of Neisseria gonorrhoeae in Portugal (2004-2009). *Sexually Transmitted Infections* 2010; **86**(6): 449-53.

20. Chisholm SA, Alexander S, Desouza-Thomas L, et al. Emergence of a Neisseria gonorrhoeae clone showing decreased susceptibility to cefixime in England and Wales. *Journal of Antimicrobial Chemotherapy* 2011; **66**(11): 2509-12.

21. Yuan LF, Yin YP, Dai XQ, et al. Resistance to azithromycin of neisseria gonorrhoeae isolates from 2 cities in China. *Sexually Transmitted Diseases* 2011; **38**(8): 764-8.

22. Ota KV, Ng LK, Melano RG, et al. Identification of sexual networks through molecular typing of quinolone-resistant Neisseria gonorrhoeae in Ontario, Canada. *Sexually Transmitted Diseases* 2011; **38**(9): 811-4.

23. Carannante A, Latini A, Cusini M, et al. Update on antimicrobial susceptibility and genotype of Neisseria gonorrhoeae isolated in Italy. *Diagnostic Microbiology and Infectious Disease* 2012; **72**(3): 288-90.

24. Hjelmevoll SO, Golparian D, Dedi L, et al. Phenotypic and genotypic properties of Neisseria gonorrhoeae isolates in Norway in 2009: Antimicrobial resistance warrants an immediate change in national management guidelines. *European Journal of Clinical Microbiology and Infectious Diseases* 2012; **31**(6): 1181-6.

25. Cole MJ, Thomas DR, Chisholm SA, et al. Molecular epidemiology of gonorrhoea in Wales (UK). *Sexually Transmitted Infections* 2013; **89**(3): 267-72.

26. Bernstein KT, Marcus JL, Barry PM, et al. Characteristics of males infected with common neisseria gonorrhoeae sequence types in the gonococcal isolate surveillance project, san francisco, California, 2009. *American Journal of Epidemiology* 2013; **178**(8): 1289-95.

27. Chen CC, Yen MY, Wong WW, et al. Tracing subsequent dissemination of a cluster of gonococcal infections caused by an ST1407-related clone harbouring mosaic penA alleles in Taiwan. *Journal of Antimicrobial Chemotherapy* 2013; **68**(7): 1567-71.

28. Ison CA, Town K, Obi C, et al. Decreased susceptibility to cephalosporins among gonococci: Data from the Gonococcal Resistance to Antimicrobials Surveillance Programme (GRASP) in England and Wales, 2007-2011. *The Lancet Infectious Diseases* 2013; **13**(9): 762-8.

29. Chisholm S, Unemo M, Quaye N, et al. Molecular epidemiological typing within the European Gonococcal Antimicrobial Resistance Surveillance Programme reveals predominance of a multidrug resistant clone. *Euro Surveill* 2013; **18**(3): 20358.

30. Singh AE, Gratrix J, Read R, et al. Neisseria gonorrhoeae multiantigen sequence typing is beneficial in further characterizing gonococcal populations in Alberta, Canada. *Sexually Transmitted Diseases* 2013; **40**(9): 744-50.

31. Horn NN, Kresken M, Korber-Irrgang B, et al. Antimicrobial susceptibility and molecular epidemiology of Neisseria gonorrhoeae in Germany. *International Journal of Medical Microbiology* 2014; **304**(5-6): 586-91.

32. Carannante A, Renna G, Conte ID, et al. Changing antimicrobial resistance profiles among neisseria gonorrhoeae isolates in Italy, 2003 to 2012. *Antimicrobial Agents and Chemotherapy* 2014; **58**(10): 5871-6.

33. Jeverica S, Golparian D, Maticic M, Potocnik M, Mlakar B, Unemo M. Phenotypic and molecular characterization of Neisseria gonorrhoeae isolates from Slovenia, 2006-12: Rise and fall of the multidrug-resistant NG-MAST genogroup 1407 clone? *Journal of Antimicrobial Chemotherapy* 2014; **69**(6): 1517-25.

34. Cheng CW, Li LH, Su CY, Li SY, Yen MY. Changes in the six most common sequence types of Neisseria gonorrhoeae, including ST4378, identified by surveillance of antimicrobial resistance in northern Taiwan from 2006 to 2013. *Journal of Microbiology, Immunology and Infection* 2015.

35. Stevens K, Zaia A, Tawil S, et al. Neisseria gonorrhoeae isolates with high-level resistance to azithromycin in Australia. *Journal of Antimicrobial Chemotherapy* 2015; **70**(4): 1267-8.

36. Chen SC, Yin YP, Dai XQ, Unemo M, Chen XS. First nationwide study regarding ceftriaxone resistance and molecular epidemiology of Neisseria gonorrhoeae in China. *Journal of Antimicrobial Chemotherapy* 2016; **71**(1): 92-9.

37. Foster K, Cole M, Hotonu O, et al. How to do it: lessons identified from investigating and trying to control an outbreak of gonorrhoea in young heterosexual adults. *Sex Transm Infect* 2016; **92**(5): 396-401.

38. Ni C, Xue J, Zhang C, Zhou H, van der Veen S. High prevalence of Neisseria gonorrhoeae with high-level resistance to azithromycin in Hangzhou, China. *Journal of Antimicrobial Chemotherapy* 2016; **71**(8): 2355-7.

39. Lahra MM, Ward A, Trembizki E, et al. Treatment guidelines after an outbreak of azithromycin-resistant Neisseria gonorrhoeae in South Australia. *The Lancet Infectious Diseases* 2017; **17**(2): 133-4.

40. Serra-Pladevall J, Barberá MJ, Callarisa AE, Bartolomé-Comas R, Andreu A. Differences in Neisseria gonorrhoeae population structure and antimicrobial resistance pattern between men who have sex with men and heterosexuals. *Epidemiology and Infection* 2017; **145**(2): 379-85.

41. Perez-Losada M, Crandall KA, Bash MC, Dan M, Zenilman J, Viscidi RP. Distinguishing importation from diversification of quinolone-resistant Neisseria gonorrhoeae by molecular evolutionary analysis. *BMC Evolutionary Biology* 2007; **7**(84).

42. Trembizki E, Wand H, Donovan B, et al. The Molecular Epidemiology and Antimicrobial Resistance of Neisseria gonorrhoeae in Australia: A Nationwide Cross-Sectional Study, 2012. *Clinical Infectious Diseases* 2016; **63**(12): 1591-8.

43. Grad YH, Kirkcaldy RD, Trees D, et al. Genomic epidemiology of Neisseria gonorrhoeae with reduced susceptibility to cefixime in the USA: A retrospective observational study. *The Lancet Infectious Diseases* 2014; **14**(3): 220-6.

44. Demczuk W, Lynch T, Martin I, et al. Whole-genome phylogenomic heterogeneity of Neisseria gonorrhoeae isolates with decreased cephalosporin susceptibility collected in Canada between 1989 and 2013. *Journal of Clinical Microbiology* 2015; **53**(1): 191-200.

45. Chisholm SA, Wilson J, Alexander S, et al. An outbreak of high-level azithromycin resistant Neisseria gonorrhoeae in England. *Sexually Transmitted Infections* 2016; **92**(5): 365-7.

46. De Silva D, Peters J, Cole K, et al. Whole-genome sequencing to determine transmission of Neisseria gonorrhoeae: an observational study. *The Lancet Infectious Diseases* 2016; **16**(11): 1295-303.

47. Didelot X, Dordel J, Whittles LK, et al. Genomic analysis and comparison of two gonorrhea outbreaks. *mBio* 2016; **7**(3).

48. Grad YH, Harris SR, Kirkcaldy RD, et al. Genomic epidemiology of gonococcal resistance to extended-spectrum cephalosporins, macrolides, and fluoroquinolones in the United States, 2000-2013. *Journal of Infectious Diseases* 2016; **214**(10): 1579-87.

49. Jacobsson S, Golparian D, Cole M, et al. WGS analysis and molecular resistance mechanisms of azithromycin-resistant (MIC &gt;2 mg/L) Neisseria gonorrhoeae isolates in Europe from 2009 to 2014. *Journal of Antimicrobial Chemotherapy* 2016; **71**(11): 3109-16.
